# A Coiled-Coil Nucleotide-Binding Domain Leucine-Rich Repeat Receptor Gene *MeRPPL1* Plays a Role in the Replication of a Geminivirus in Cassava

**DOI:** 10.3390/v16060941

**Published:** 2024-06-11

**Authors:** Elelwani Ramulifho, Chrissie Rey

**Affiliations:** 1 Plant Biotechnology Laboratory, School of Molecular and Cell Biology, University of the Witwatersrand, Johannesburg 2001, South Africa; ramulifhoelelwani@gmail.com; 2 Germplasm Development, Agricultural Research Council, Small Grain Institute, Bethlehem 9700, South Africa

**Keywords:** NLR, geminivirus, SACMV, cassava mosaic disease, *MeRPPL1*, CRISPR/Cas9, resistance gene, TME3, tolerance

## Abstract

Disease resistance gene (R gene)-encoded nucleotide-binding leucine-rich repeat proteins (NLRs) are critical players in plant host defence mechanisms because of their role as receptors that recognise pathogen effectors and trigger plant effector-triggered immunity (ETI). This study aimed to determine the putative role of a cassava coiled-coil (CC)-NLR (CNL) gene *MeRPPL1* (*Manes.12G091600*) (single allele) located on chromosome 12 in the tolerance or susceptibility to South African cassava mosaic virus (SACMV), one of the causal agents of cassava mosaic disease (CMD). A transient protoplast system was used to knock down the expression of *MeRPPL1* by clustered regularly interspaced short palindromic repeats-CRISPR-associated protein 9 (CRISPR-Cas9). The *MeRPPL1*-targeting CRISPR vectors and/or SACMV DNA A and DNA B infectious clones were used to transfect protoplasts isolated from leaf mesophyll cells from the SACMV-tolerant cassava (*Manihot esculenta*) cultivar TME3. The CRISPR/Cas9 silencing vector significantly reduced *MeRPPL1* expression in protoplasts whether with or without SACMV co-infection. Notably, SACMV DNA A replication was higher in protoplasts with lower *MeRPPL1* expression levels than in non-silenced protoplasts. Mutagenesis studies revealed that protoplast co-transfection with CRISPR-*MeRPPL1* silencing vector + SACMV and transfection with only SACMV induced nucleotide substitution mutations that led to altered amino acids in the highly conserved MHD motif of the *MeRPPL1*-translated polypeptide. This may abolish or alter the regulatory role of the MHD motif in controlling R protein activity and could contribute to the increase in SACMV-DNA A accumulation observed in *MeRPPL1*-silenced protoplasts. The results herein demonstrate for the first time a role for a CNL gene in tolerance to a geminivirus in TME3.

## 1. Introduction

Cassava (*Manihot esculenta* Crantz) serves as both an industrial crop and a staple food source for more than 800 million people worldwide [1]. However, its production is threatened by viruses, belonging to the genus *Begomovirus* within the *Geminiviridae* family [2], that cause cassava mosaic disease (CMD) [3]. Cassava mosaic begomoviruses (CMBs) rank among the most damaging viruses, causing losses of at least 25 million tons annually in Africa, India, and Sri Lanka [4,5]. Amongst several CMB species is the South African cassava mosaic virus (SACMV), which is a bipartite (DNA A and B) circular ssDNA begomovirus [6]. CMD has effectively resulted in yield losses of up to 82% [4] by significantly reducing the number and size of tubers, depending on factors such as virus virulence, the susceptibility or tolerance/resistance of the host, and other environmental factors [7]. Infection presents as deformed/curly leaves with a yellow/pale-green mosaic and general reduced plant growth. Conventional strategies, such as phytosanitation, cultural practices, and whitefly vector control for controlling CMD have failed over the past years [5]. However, breeding programs have successfully generated cassava genotypes with some tolerance/resistance to CMD, exhibiting higher yields compared to susceptible varieties [8]. However, breeding programs have successfully generated cassava genotypes with some tolerance/resistance to CMD, exhibiting higher yields compared to susceptible genotypes [8]. Three cassava QTL markers (CMD1, CMD2, and CMD3) are associated with conferring resistance/partial resistance to CMD. Tropical *M. esculenta* (TME) [9,10] exhibits CMD2-associated monogenic dominant resistance. Landraces/cultivars with this marker display near-immunity phenotypes against most cassava geminiviruses [11]. Rabbi et al. [10] mapped the dominant monogenic resistance CMD2 locus and reported that all markers linked to qualitative resistance occur in the same chromosome region. Further genome-wide association studies (GWAS) predicted the location of genes associated with CMD2 on chromosome 8 and further identified two possible epistatic loci and multiple resistance alleles at the major CMD2 QTL [12]. The update of the cassava genome assembly from version *Manihot esculenta* v4.1 to v6.1/v8.1 relocated the CMD2 QTL on chromosome 12 (https://phytozome.jgi.doe.gov, accessed on 8 January 2024). 

While recent mapping studies identified the CMD2 QTL on chromosome 12, the actual gene(s) involved in triggering CMD2-associated immunity, and their associated molecular mechanisms, remain unknown. Plant immunity to viral pathogens comprises three major mechanisms, namely pathogen-triggered immunity (PTI), effector-triggered immunity (ETI) and innate immunity (RNA silencing) [13]. Monogenic dominant resistance is often associated with ETI triggered by resistance genes. These resistance (R) genes do not always but often encode nucleotide-binding leucine-rich repeat (NLR) protein receptors that are critical players as pathogen effector recognition receptors. NLRs perceive virus effectors and trigger the activation of signal transduction cascades resulting in ETI, suggesting their crucial role in defence response mechanisms [14,15]. 

Resistance genes associated specifically with plant viruses were reported in an overall review by Sett et al. [16]. Specifically, those induced by geminiviruses are *CYR1*, associated with mung bean yellow mosaic India virus (MYMIV) [17]; *Ty-2*, associated with tomato yellow leaf curl virus (TYLCV) [18,19]; *Sw-5a*, associated with tomato leaf curl New Delhi virus [20]; and *Ty-6*, associated with tomato yellow leaf curl virus and tomato mottle virus [21]. While a dominant monogenic resistance locus *CMD2/CMD3* is reported for cassava mosaic geminiviruses [9], no specific genes specifically associated with this QTL have been confirmed in vivo. Intracellular R gene-translated proteins are represented mainly by the NLR receptors [16]. The natural *Ty-2* gene that confers high resistance to the geminivirus tomato yellow leaf curl virus (TYLCV) encodes an NLR protein named TYNBS1, which activates ETI via the recognition of a *Rep/C1* gene-encoded geminiviral effector [18,19]. More recently, a nonsynonymous single nucleotide polymorphism in *DNA polymerase δ subunit 1* (*MePOLD1*) located within this CMD2 region has also been linked to CMD2-type resistance. Virus-induced gene silencing of *MePOLD1* in a CMD-susceptible cassava variety produced a recovery phenotype typical of CMD2-type resistance [22]. 

Despite progress in cassava breeding for CMD resistance, specific cassava NLR genes involved in the molecular mechanisms of immunity/tolerance have not been identified or experimentally demonstrated. While several NLR resistance gene analogues (RGAs) were identified in seven African cassava landraces [23], no in planta functional studies on these were performed. The cassava transcriptome [24], proteome [25], and sequenced cassava genome data [26,27] revealed putative R genes/proteins that could be involved in the recognition of CMD-associated geminiviral pathogens. Furthermore, an NLR resistance gene with a single allele, *MeRPPL1*, recently relocated to cassava chromosome 12, was suggested to be involved in CMD tolerance in cassava cultivar TME3 [28]. From all the identified NLR genes with the potential to confer resistance to viruses, only a handful have been cloned and functionally characterised in different plants, including the orphan crop cassava. This research aimed to ascertain whether *MeRPPL1* is associated with tolerance to SACMV in CMD-tolerant TME3 protoplasts. Clustered regularly interspaced short palindromic repeats (CRISPR) editing of *MeRPPL1* was performed in an established cassava protoplast system. The results herein demonstrate, for the first time, a role for a resistance gene *MeRPPL1* in a CMD-tolerant cassava host in response to a geminivirus.

## 2. Materials and Methods

### 2.1. MeRPPL1 Expression Knockdown gRNA Cassette Design and Synthesis

Two gRNA targets were identified from the genomic sequence of Manes.12G091600 (herein referred to as *MeRPPL1*)genomic sequence using CRISPR version 4.7 [29]. The gRNAs were used to design a duplex gRNA cassette in the following arrangement: *Arabidopsis thaliana* U6-26 promoter + pre-tRNA + crRNA 1 + SpCas9 tracrRNA + pre-tRNA + crRNA 2 + SpCas9 tracrRNA + terminator (Figure S1). The designed duplex silencing cassette was synthesised in vitro and cloned into a pBluescript II SK(+) plasmid at Inqaba Biotech (Pretoria, South Africa).

### 2.2. Cloning the CRISPR Cassettes into pCAMBIA 1380 Delivery Vector

The gRNA cassette was digested from the pBluescript II SK(+) plasmid using restriction enzymes HindIII and BglII and cloned into the delivery vector pCAMBIA 1380 to produce pC1380-*MeRPPL1*. A Cas9 cassette, consisting of *Tobacco mosaic virus* promoter + Cas9 + eGFP + Hsp terminator from the pL1m-f2-p35s-cas9-egfp-nucleo-thsp Golden Gate vector, was cloned to produce pC1380-*Cas9* (Figure S2). Cloning was performed using a 5:1 (insert:vector) molar ratio. The cassettes were ligated into delivery vectors using the T4 DNA ligase enzyme (ThemoFisher Scientific, Waltham, MA, USA) according to the manufacturer’s manual, then transformed into chemically competent *E. coli* DH5-alpha cells using the snap freeze method. Restriction enzyme (HindIII and BglII) digestions and sequencing confirmed the positive ligation of the cassettes into the delivery vector. 

### 2.3. CRISPR-Mediated MeRPPL1 Expression Knockdown: Protoplast Isolation and Transfection

Methods with minor modifications were based on a previous study where the TME3 protoplast-SACMV system was optimised [30]. Protoplasts were isolated from 4-week-old leaf mesophyll cells of cassava TME3 plantlets propagated in vitro on Murashige and Skoog (MS) medium [31] by the shoot multiplication technique in sterile tissue culture bottles in environmentally controlled growth conditions (16:8 h (light: dark); 26–28 °C). Three independent biological replicate experiments were performed. For the first step, approximately 500 mg of fully expanded leaves were transversely sliced into thin strips and immediately plasmolysed in 10 mL CPW9M medium in order to facilitate subsequent cell wall degradation to release the protoplasts [30]. This CPW9M medium is composed of 0.5 M mannitol, 27.2 mg KH_2_PO_4_, 100 mg KNO_3_, 150 mg CaCl_2_, 250 mg MgSO_4_, 2.5 mg Fe_2_(SO_4_)_3_.6H_2_0, 0.6 mg KI, 0.00025 mg CuSO_4_ per litre; pH 5.8. Incubation was performed for one hour at room temperature. CPW9M medium was replaced with 10 mL enzyme solution (5 mM MES, 2-(N-morpholino) ethanesulfonic acid, MES, 0.4% cellulase, 0.2% macerozyme) before vacuum infiltrating the leaf strips at 80 kPa for 30 min (step 2). The mixture was left to gently shake at 40 rpm for 24 h (overnight) in the dark at room temperature. Protoplasts were then released (step 3) by shaking at 80 rpm for 5 min, followed by filtering through a 75 μm sieve into a 50 mL round-bottom tube. Protoplasts were collected by centrifuging at 100 g for 10 min, washed twice with CPW9M medium, and resuspended in MMg solution (0.4 M mannitol, 15 mM MgCl_2_, 4 mM MES, pH 5.8) (step 4). 

The integrity and viability of protoplasts at 24 h after steps 3 and 4 were assessed using the Olympus BX 63 OM/FM microscope (Olympus Scientific Solutions, Waltham, MA, USA) and Evans Blue dye staining, respectively. Protoplasts were quantified using the BD Accuri^TM^ C6 flow cytometer (BD Biosciences, Franklin Lakes, NJ, USA). One hundred and fifty protoplasts were counted for viability prior to treatments (at 24 h post-treatment; step 4) from the three independent biological experiments. On average, 135 out of 150 (90%) counted protoplasts were viable and intact. The ratio of viable to non-viable protoplasts was 90:10. 

Three independent biological experiments (*n* = 3) were performed, and for each biological experiment, three technical replicates were performed. For each treatment, the protoplasts were diluted to 10^4^ protoplasts/mL. Protoplasts were transfected with (i) 15 μg of the pCAMBIA 1380-derived CRISPR vectors only (pC1380-MeRPPL1 + pC1380-Cas9); (ii) CRISPR vectors and 4 μg of each of the pBIN19-SACMV-DNA A and pBIN19-SACMV-DNA B infectious clones [32]; and (iii) SACMV-A and B infectious clones only. Control protoplasts were non-transfected or transfected with the pCAMBIA1380 vector only (mock). Previous well-established infectivity studies with SACMV infectious clones in planta [6] and a previous study in TME3 protoplasts [30] demonstrated that pBIN9 has no effect on cassava and was not included as an additional control. Polyethylene glycol 4000 (PEG4000) to a final concentration of 25% was immediately added to the transfected protoplasts and incubated at 28 °C for 20 min. The transfection mixture was then diluted with three volumes of W5 solution (154 mM NaCl, 125 mM CaCl_2_, 5 mM KCl, 2 mM MES, pH 5.8) and washed twice by centrifuging at 100× *g* for 2 min. Protoplasts were resuspended in 300 μL of WI solution (4 mM MES, 0.5 M mannitol, 20 mM KCl, pH 5.8) and incubated in the dark overnight at 28 °C to induce gene expression. Protoplasts were collected by centrifuging at 100× *g* for 5 min before being used in subsequent experiments. The viability of the protoplasts at 24 hpt was in a similar range for all transfections [(i) the CRISPR silencing vector only control (RC), (ii) SACMV (S), or both virus and CRISPR vector (RCS); pCAMBIA1350 only (mock)].

### 2.4. RNA Extraction and RT-qPCR Gene Expression Analysis

RNA was extracted from protoplasts at 24 hpt using QIAzol Lysis Reagent (QIAGEN) and cleaned using GeneJET RNA Cleanup and Concentration Micro Kit according to the manufacturer’s protocol. RevertAid First Strand cDNA Synthesis Kit (ThermoFisher Scientific) was used to synthesise first-strand cDNA using an Oligo(dT)18 primer, according to the manufacturer’s protocol. For relative *MeRPPL1* expression, RT-qPCR was performed in triplicate with cDNA as a template using Maxima SYBR Green/ROX qPCR Master Mix (2X) with optimised *MeRPPL1* primers (Fwd: 5′ agctcttgtgtatgggtgcc 3′, Rev: 5′ atggtgcccataatggaggc 3′). Log 2 fold change in the expression of *MeRPPL1* was normalised to *18S rRNA* gene [29]. Basal MeRPPL1 expression level was measured in non-transfected cassava TME3 protoplasts and protoplasts following transfection with SACMV, the CRISPR silencing vector, and the CRISPR + SACMV infectious clones. Expression was relative to mock, which was the pCAMBIA1380 vector only. Gene expression fold change was calculated using the log 2^(ΔΔCT)^ method [33]. Student’s *t*-test was used to assess statistical differences in *MeRPPL1* expression between treatments.

### 2.5. DNA Extraction and Viral Replication Quantification

DNA was extracted from transfected protoplasts using a User-Developed Protocol to Isolate Genomic DNA from the Interphase and Organic (phenol) Phase of Tissue Samples Treated with QIAzol Lysis Reagent (QIAGEN, Germantown, MD, USA). The DNA was cleaned up using Thermo Scientific GeneJET Gel Extraction and DNA Cleanup Micro Kit according to the manufacturer’s protocol before it was fast digested with the restriction enzyme *DpnI* (ThermoFisher Scientific, Massachusetts, USA). The quality of the *Dpn-I* digested DNA was visually checked by gel electrophoresis (1.5% agarose gel) to confirm that only newly replicated SACMV DNA A was measured and not input DNA from the transfection. 

Twenty-five nanograms of the digested DNA was used as a template for SACMV DNA A relative viral load quantification using qPCR. Quantification was performed in triplicates using Maxima SYBR Green/ROX qPCR Master Mix (2X) (ThermoFisher Scientific, Massachusetts, USA) with SACMV coat protein (CP) (on DNA A) primers (Fwd: 5′ acgtccgtcgcaagtac 3′, Rev: 5′ attgtcatgtcgaatagtacg 3′). As optimised in previous cassava studies [23,29], *18S rRNA* was used as a reference gene for qPCR quantifications. The SACMV viral load was calculated using the log 2^(ΔΔCT)^ method [33]. Student’s *t*-test was used to assess statistical differences between samples.

### 2.6. Sequencing, Mutagenesis Analysis, and Protein Structure Prediction

*DpnI*-digested DNA samples from the treatments (Section 2.3) were sent for the sequencing of *MeRPPL1* at Inqaba Biotech (Pretoria, South Africa) using the ABI 3500XL Genetic Analyzer. SnapGene software was used to assess sequence variations (https://www.snapgene.com/, accessed on 8 January 2024), while MEGAX software (version 2.0) was used for multiple sequence alignment using the ClustalW algorithm [34]. Target gene homologs from wild-type AM560-2 (Accession NC_035172.1) and *Manihot esculenta* cultivar TME3 (RefSeq ID RSFT01000007, GenBank ID GCA_003957995.1, unpublished data) were used as references for the alignments. The frequency of clones with the altered sequence was obtained by expressing the number of amplicons from the polyclonal mix with sequence alteration as a ratio of 10 amplicons sequenced.

AlphaFold.ipynb (https://colab.research.google.com/, accessed on 8 January 2024) [35] was used to predict the secondary structure of the proteins of translated amino acids from the sequenced DNA results. The monomer model was used for individual peptides. The run_relax option was selected to run AlphaFold and generate the predicted protein structures. 

## 3. Results

### 3.1. MeRPPL1 gRNA Cassette Confirmation

**Figure 1:**
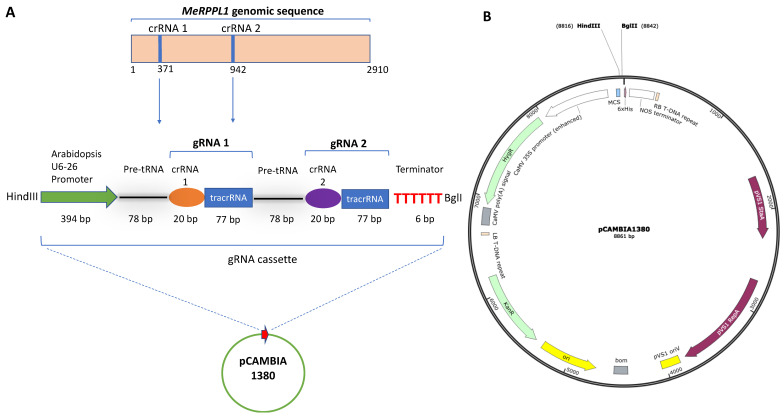
(**A**) *MeRPPL1* gene (2910 nt), illustrating the positions (371 nt and 942 nt) for the design of the CRISPR silencing constructs crRNA1 and crRNA2. Below: Representation of the *MeRPPL1* gRNA silencing cassette (760 bp): (L to R) the Arabidopsis U6-26 promoter; gRNA 1 and gRNA2 silencing constructs and terminator. Each gRNA is composed of a pre-tRNA, crRNA (20 bp), and tracrRNA (77 bp) region. The 5′ and 3 have a HindIII and BglI restriction site, respectively. (**B**) pCAMBIA 1380 vector (8861 bp) showing restriction sites HindIII and BglI where the *MeRPPL1* gRNA cassette was cloned into.

The *MeRPPL1* gene is a single allele which was shown to reside in a locus on a chromosome 12 scaffold which is also associated with *CMD2*-dominant resistance in cassava (Figure S3). It was also selected from the transcriptomic data of the CMD-tolerant cassava cultivar TME3 infected with SACMV. Upregulation was observed at 32 days post-infection (dpi) [23], suggesting a putative role in response to SACMV. Phytozome (https://phytozome-next.jgi.doe.gov/report/gene/Mesculenta_v8_1/Manes.12G091600, accessed on 8 January 2024) was accessed in January 2021, to retrieve the *MeRPPL1* sequence [phytozome genome ID: 305; NCBI taxonomy ID: 3983 A Pathogen Receptor Genes (PRGs) database (PRGdb) version 3.0 (http://www.prgdb.org/prgdb/plants/?id=375, accessed on 8 January 2024)] to confirm that *MeRPPL1* is a nucleotide-binding leucine-rich repeat (NLR) gene. The *MeRPPL1* gRNA cassette (Figure 1A) was cloned into a pCAMBIA 1380 vector (Figure 1B) and confirmed by restriction enzyme digestion and Sanger sequencing. The vector was digested with HindIII and BglII, resulting in the expected band of approximately 760 bp by gel electrophoresis.

### 3.2. Protoplast Isolation and Viability

**Figure 2:**
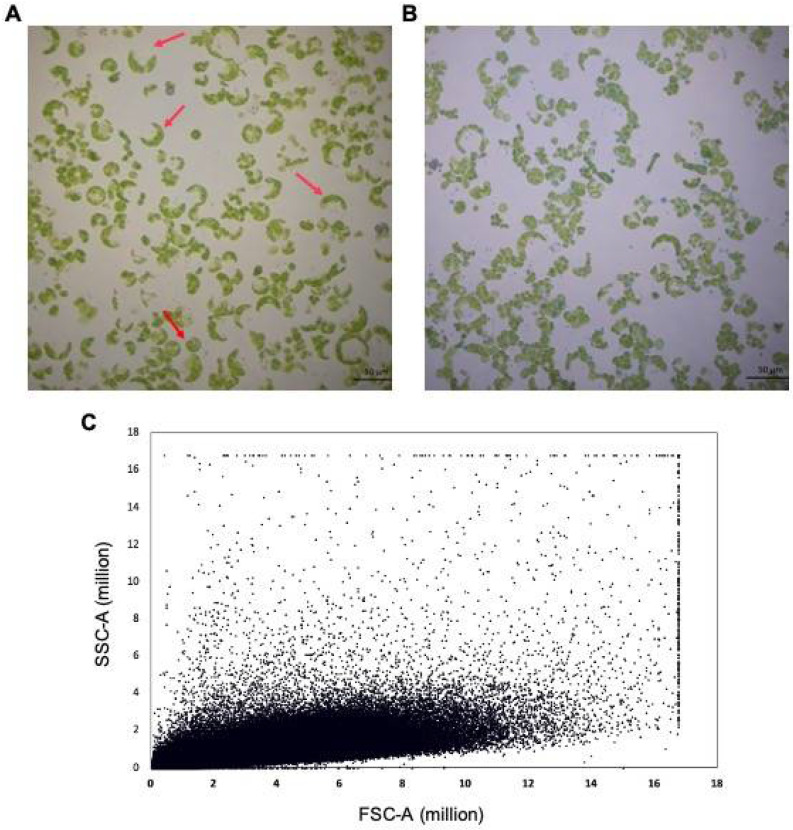
Analyses of the quality and quantity of protoplasts isolated from cassava TME3 landrace. (**A**) Cassava protoplasts isolated from leaf mesophyll cells. Red arrows show intact spherical protoplasts with chloroplasts around the edge of the cell membrane. (**B**) Evans Blue dye protoplast viability assessment on isolated protoplasts visualised under a light microscope. Viable cells do not take up the blue dye, while non-viable cells take up the blue dye due to compromised cell membranes (leaky). Hence, since the protoplasts do not appear blue (only the background appeared pale blue from the dye), this demonstrates that the protoplasts are viable. The scale bar on protoplast images is 50 μm. (**C**) Protoplasts’ cell count and quality were measured using flow cytometry density measurement based on side scatter (SSC-A) and forward scatter (FSC-A).

Protoplasts were isolated from 4-week-old leaf mesophyll cells of cassava TME3 plantlets propagated in vitro by the shoot multiplication technique in sterile tissue culture bottles in environmentally controlled conditions. When analysed under a light microscope, spherical intact and irregularly shaped protoplasts were observed (Figure 2A). The diameters between 16.1 and 38.9 μm were measured from at least ten spherical protoplasts. Chloroplasts were observed around the edge of protoplasts’ cell membranes. Before transfection, protoplasts were assessed for viability using Evans Blue dye, and at least 90% were viable (Figure 2B). On average, 135 out of 150 counted untreated protoplasts were viable and intact (ratio 90 viable:10 non-viable). The average viability of the protoplasts at 24 hpt was in a similar range (~90%) after all transfections [(i) pCAMBIA1350; SACMV only (pBIN19-SACMV-DNA A and pBIN19-SACMV-DNA B infectious clones); CRISPR vector pC1380-*MeRPPL1* + pC1380-*Cas9* only; and CRISPR vector + SACMV)]. The transfection rate (90%) was similar to a previous study in TME3 protoplasts [90%].

In addition, flow cytometry was used to measure the integrity of protoplasts depending on their forward scatter (FSC) and side scatter (SSC) gating [36], and a 3.8 × 10^5^/μL cell count was recorded (Figure 2C).

### 3.3. Expression of MeRPPL1 in CRISPR/Cas9-Treated Protoplasts

**Figure 3:**
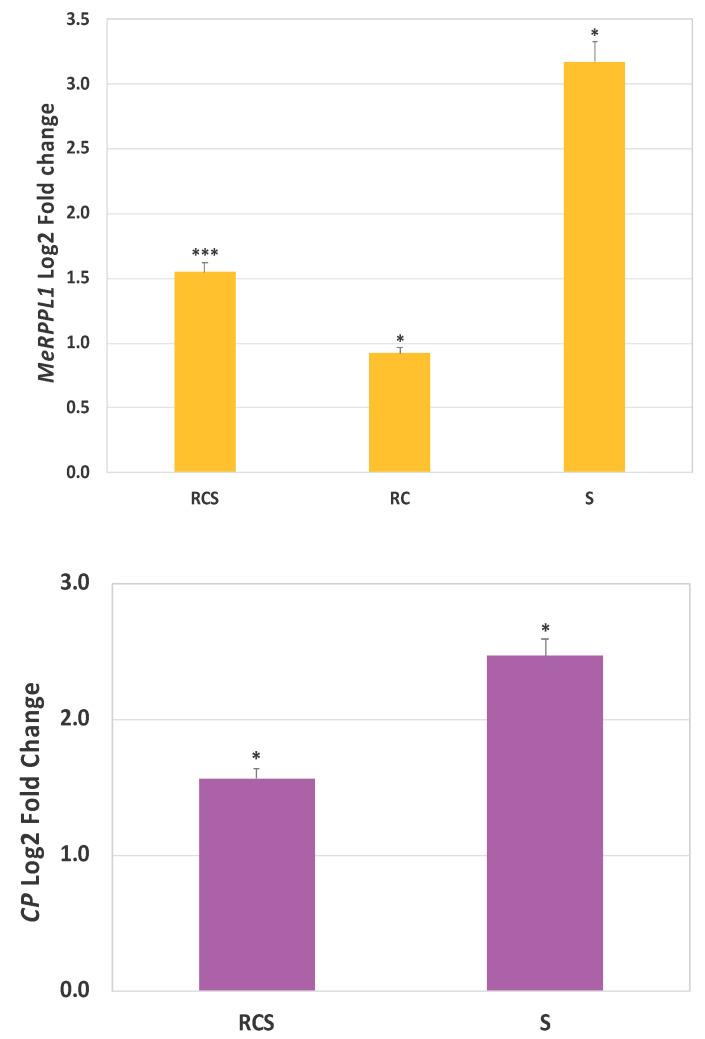
**Above (top):** log 2 fold change in expression of MeRPPL1 normalised to 18S rRNA gene in cassava TME3 protoplasts following transfection with the CRISPR vectors. Expression was relative to mock which was the pCAMBIA1380 vector only. **Below (bottom)**: Quantification of SACMV measured as the log2 fold change in the CP gene (on DNA A) following CRISPR knockdown of MeRPPL1 expression. S = TME3 protoplasts transfected with SACMV only (pBIN19-SACMV-DNA A and pBIN19-SACMV-DNA B infectious clones); RC = CRISPR vector pC1380-MeRPPL1 + pC1380-Cas9 only; RCS = CRISPR vector + SACMV. Error bars represent means ± S.D. (*n* = 3). One and three asterisks indicate statistically significant differences between treated samples at *p* ≤ 0.05 and 0.001, respectively, using Student’s *t*-test.

In this study, RT-qPCR was performed at 24 h post-transfection (hpt). To measure the relative expression levels of CRISPR-targeted *MeRPPL1* in TME3 protoplasts, real-time qPCR was performed on cDNA using *MeRPPL1* primers, with the *18S rRNA* gene for normalisation. *MeRPPL1* expression in non-transfected leaf protoplasts was low (log2fold −9.8). RT-qPCR comparisons in expression between transfection with CRISPR vectors only (RC), CRISPR vectors and SACMV (RCS), and SACMV only (S) were undertaken (Figure 3 top). The highest log2 fold change (3.1 log2 fold increase) of *MeRPPL1* expression normalised to 18S *rRNA* was recorded in protoplasts that were only transfected with SACMV infectious clones (S), suggesting that SACMV triggers a *MeRPPL1* response in SACMV-tolerant TME3-derived protoplasts. 

A decrease (log2 fold 1.5) in *MeRPPL1* expression was observed in protoplasts co-transfected with both the CRISPR/Cas9 vectors and SACMV infectious clones (RCS) compared to protoplasts transfected only with SACMV (S) (Figure 3 top). While the expression of *MeRPPL1* was not abolished, its expression was significantly down-regulated by CRISPR. These results showed that SACMV may play a role in reducing/mitigating the impact of the CRISPR vectors on *MeRPPL1* expression. This is further supported by the fact that the transfection of protoplasts with CRISPR vectors (RC) only, without SACMV, experienced the lowest (log2-fold = 0.9) *MeRPPL1* gene expression knockdown compared to SACMV-transfected (S) protoplasts and CRISPR vectors + SACMV (RCS). Alternatively, it may well be that SACMV’s ability to upregulate the expression of *MeRPPL1* remains even when CRISPR is acting to reduce it.

### 3.4. South African Cassava Mosaic Virus Replication Quantification

Relative log2 fold change in SACMV replication (viral load) was measured by qPCR of the coat protein gene (AC1) on DNA A at 24 h post-transfection using the log 2^(ΔΔCT)^ method (Figure 3 below). Protoplasts that were transfected with only the pBIN19-SACMV-DNA A and pBIN19-SACMV-DNA B infectious clones (S) had significantly higher (2.5-fold) SACMV DNA A accumulation in comparison to co-inoculation with the pC1380-pC1380-Cas9 vector (RC) + SACMV infectious clones (S) (RCS), where transfected protoplasts had a significantly (*p* ≤ 0.05) lower (1.6-fold) SACMV viral load.

### 3.5. CRISPR/Cas9-Mediated Mutations in MeRPPL1

**Figure 4:**
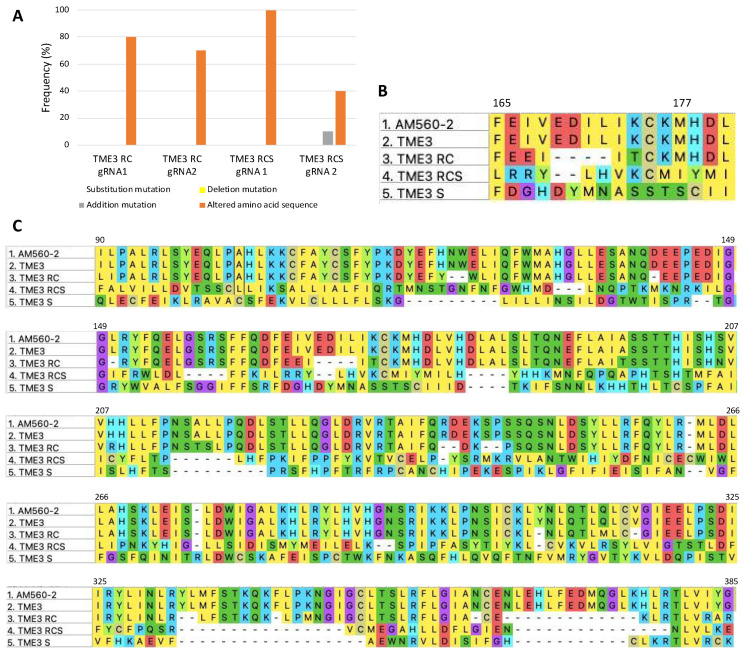
(**A**) A summary of the types of mutations observed in MeRPPL1 in the SACMV-tolerant TME3 cultivar, and their frequency (derived from sequencing 10 amplicons per genotype), induced by gRNA1 or gRNA2 in RC (silencing vector) or RSC (silencing vector + SACMV)-treated protoplasts. Random substitution mutations resulting in amino acid changes occurred the most frequently (80–100% following gRNA1 treatment and 40–65% with gRNA2), while deletion mutations (yellow bar below graph) were not observed. Addition mutations (10% frequency) were only observed in TME3 RCS protoplasts treated with gRNA2. (**B**) Nucleotide mutations resulting in amino acid mutations in the conserved MHD domain (position 165–180) induced by CRISPR vector (RC), RC + SACMV infectious clones-edited MeRPPL1 (TME3 RCS), and SACMV-infected only (TME3 S). Mutations include substitutions and deletions. (**C**) Amino acid (aa) sequence alignment of MeRPPL1 in cassava TME3 cultivar protoplasts subjected to the different treatments. Amino acid deletions and substitutions were most frequently observed. Mutations in MeRPPL1 were determined by aligning the sequences of treated samples to MeRPPL1 homologs from wild-type reference genome AM560-2 and TME3 (GenBank ID GCA_003957995.1) using the ClustalW algorithm in MEGA X software (version 2.0).

*DpnI*-treated DNA extracted from transfected protoplasts was sent to Inqaba Biotech (Pretoria, South Africa) for sequencing of the *MeRPPL1* gene in order to determine nucleotide mutations induced by CRISPR, and consequently, the derived translated amino acid (aa) sequence alterations. This is critical, as altered amino acids would lead to changes in the folding/secondary structure of the MeRPPL1 protein, affecting its interaction with SACMV effectors. The sequencing results of ten amplicons from a polyclonal mix revealed different nucleic acid mutations caused by the CRISPR/Cas9 vectors. Substitution mutations occurred randomly at a 90–100% frequency, while addition mutations upstream of *MeRPPL1* crRNA2 in pC1380-*MeRPPL1* + pC1380-*Cas9* + SACMV (TME3 RCS) samples occurred at a 10% frequency (Figure 4A). These nucleotide mutations resulted in 40–100% frequencies where amino acid sequences were altered. Interestingly, mutations in both TME3 transfected with the CRISPR silencing vector and SACMV (TME3 RCS) and SACMV only (TME3 S) led to amino acid changes in the MHD domain (from position 177) of the MeRPPL1 peptide, where methionine (M); histidine (H); and aspartic acid (D) amino acids were substituted with isoleucine (I); tyrosine (Y) or methionine (M); and serine (S), cysteine (C), or isoleucine (I), respectively. MeRPPL1 translated peptide from only the CRISPR vector-transfected protoplasts (TME3 RC) retained the conserved MHD domain (Figure 4B). From the examination of the entire *MeRPPL1* sequenced region, it was evident that substitution mutations occurred randomly throughout the gene region leading to several changes in amino acid sequences of the peptide when compared to the peptide sequence of the wild-type reference genome AM560-2 and untreated TME3 MeRPPL1 homologs (Figure 4C). Interestingly, the infection of TME3 protoplasts with only SACMV also resulted in random substitution mutations that altered genes and translated peptide sequences.

**Figure 5:**
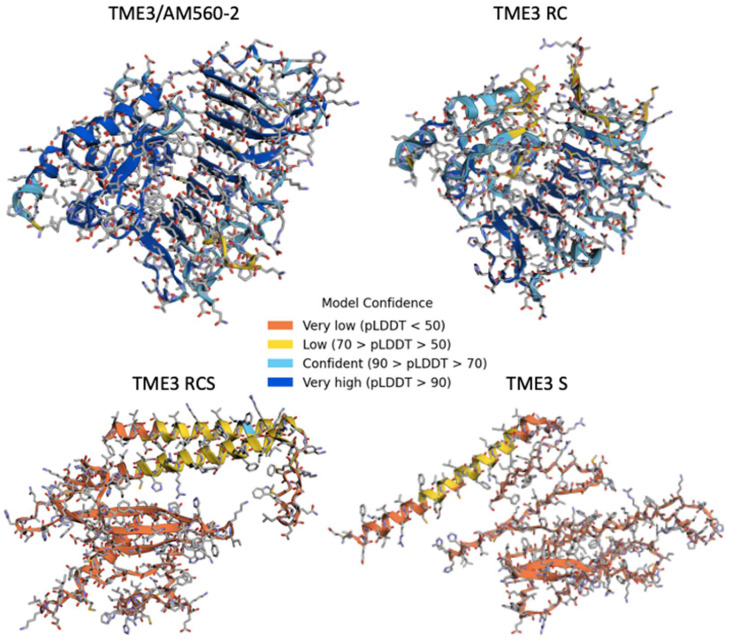
MeRPPL1 protein secondary structure predictions derived from deduced amino acids. The structures were predicted using translated amino acid sequences from the Sanger sequencing results and alignment in Figure 4C. The reference sequence is the MeRPPL1 homolog from wild-type AM560-2 and TME3 (GenBank ID GCA_003957995.1). TME3 RC = TME3 protoplasts transfected with CRISPR vectors only. TME3 RCS = TME3 protoplasts co-transfected with CRISPR vectors + SACMV infectious clones. TME3 S = TME3 protoplasts transfected with SACMV infectious clones. pLDDT corresponds to the model’s predicted score and is used to colour-code the residues of the predicted model in the 3D structure viewer [37]. pLDDT > 90 are expected to be modelled to high accuracy; between 70 and 90 are expected to be modelled well; between 50 and 70 are low confidence; less than 50 is a reasonably strong predictor of disorder.

Since alterations in amino acids affect the secondary structure of proteins, and hence their function, amino acid sequences (from Figure 4C) were used to predict secondary protein structures of MeRPPL1 following the different treatments, with reference to wild-type AM560-2 and TME3 MeRPPL1 homologs, using AlphaFold.ipynb (Figure 5; Figures S4–S7). CRISPR-induced mutations in amino acids affected the secondary structure of the MeRPPL1 predicted protein to varying levels, resulting in model prediction scores between very low (pLDDT < 50) and very high (pLDDT > 90) [37]. MeRPPL1 from TME3 RC displayed a secondary structure more similar to that of the AM560-2/TME3 reference protein compared to TME3 RCS and TME3 S. The protein secondary structure was mostly made up of residues that were modelled to high (pLDDT between 70 and 90) and very high (pLDDT > 90) confidence levels, making it a good backbone prediction that also characterised binding sites. TME3 RCS had a few residues with good backbone prediction (pLDDT between 70 and 90). The remainder of the protein structure, and that of the TME3 S model, were dominated by regions with low and very low confidence, with pLDDT scores between 50 and 70 and less than 50, respectively. Low-confidence regions still appeared to have a backbone prediction; however, this should be interpreted cautiously. Very low confidence scores also often predict a ribbon-like structure, which can be a strong indication of protein structure disorder. This suggests that silencing CRISPR vectors + SACMV and SACMV caused mutations leading to highly disordered proteins. These mutations could potentially affect the functioning of the protein and its binding capacity to SACMV virulence factors/proteins and/or other cellular interacting proteins.

## 4. Discussion

While many studies on resistance proteins (NLRs) have been undertaken in experimental plant hosts such as *Arabidopsis thaliana* and *Nicotiana benthamiana*, and in a range of annual plants, there are few studies on NLRs in perennial crops, including cassava. Additionally, most studies have been performed on pathogen-resistant plants, not virus-tolerant hosts. Despite some recent progress [38,39,40,41], the molecular mechanisms of cassava mosaic disease (CMD) resistance or tolerance are not fully comprehended. It is reported from transcriptome/proteome data that the upregulation of R gene expression [24,28] and the overexpression of some R proteins [25] are induced upon the invasion of cassava by SACMV; however, no laboratory data have been provided to date to support these findings. This study demonstrates experimentally, for the first time in the orphan crop cassava, the involvement of a CNL-type R gene, *MeRPPL1*, in response to a geminivirus associated with CMD tolerance. The CRISPR/Cas9 vector successfully induced mutations in *MeRPPL1* in the SACMV-tolerant TME3 cultivar, resulting in a significant decrease in the expression of the *MeRPPL1* gene compared to TME3 protoplasts transfected with SACMV only. This resulted in an increase in SACMV-DNA A replication, providing evidence that this *CNL* gene is involved in the response to SACMV. Notably, the highest log2 fold increase in *MeRPPL1* expression relative to 18S *rRNA* was recorded in protoplasts that were transfected with only SACMV infectious clones (S), suggesting that SACMV triggers a significant *MeRPPL1* response in SACMV-tolerant TME3-derived protoplasts. The fact that *MeRPPL1* maps to chromosome 12 associated with the CMD2 resistance-associated QTL (Figure S3) made it an informed candidate for this study. 

A cassava protoplast system was selected, as virus DNA accumulation in cassava leaf mesophyll and tobacco BY-2 protoplasts has previously been observed from 6 to 24 h [30] and 36 to 48 h [42] post-transfection, respectively. Additionally, protoplasts lack a cell wall, making it easy for the viral DNA to enter the cell by transfusion across the cell membrane and replicate inside the host cells [43]. Furthermore, in a previous study, the stability of transient expression in TME3 protoplasts of a *MeE3 ligase* was verified by the detection of eGFP expression at 24 hpt and also showed that at least 90% of protoplasts had been successfully transformed [30]. The expression of *MeRPPL1* in this study was low in untreated cassava TME3 protoplasts, and in tomato and potato, approximately 10% of all R genes were shown to be differentially expressed (either up or down) in non-infected plants [44]. Differences in R gene expression can also be tissue-specific. In this study, young leaves just below the apical meristem of cassava TME3 were selected for protoplast generation, as they represent actively dividing cells/growth that geminiviruses target due to their replication cycle. Since NLRs are well known for their functional role as receptors for viral pathogen effectors [45,46], which induce downstream effector-triggered immunity (ETI), we propose that the knockdown of the expression of the CNL, *MeRPPL1*, negatively affected ETI, resulting in an increase in SACMV replication in cassava protoplasts. A more recent study [47] also identified a novel LRR-RLK protein that contributes to tolerance to tobacco mosaic virus (TMV) in *N. tabacum*. Interestingly, the level of this protein was significantly elevated in TMV-infected tolerant *N. tabacum* alongside an E3 ubiquitin ligase (E3L). It was also shown in SACMV-infected cassava TME3 protoplasts that an E3L was upregulated and is also involved in tolerance to SACMV [30]. The lack of studies using the CRISPR/Cas system to target plant defence response genes in protoplasts limits further comparisons. Future in planta studies will be performed to confirm the observations in this study.

The NLR gene, *Ty-2* in tomato, is among the few identified genes shown to confer resistance to begomoviruses in plants [18]. Other reported geminivirus-associated NLRs are *CRY1* (CC-NBS-LRR) and *Sw-5a* (SD-CC-NBS-LRR) in *Vigna radiate* and *Solanum lycopersicum*, respectively [16]. While *Ty-2* in tomatoes plays a role in resistance to tomato yellow leaf curl virus (TYLCV), the type of gene has not been demonstrated [16]. *MeRRPL1* is the first NLR gene reported in cassava to respond to a CMD-associated geminivirus. However, the Avr protein/effector associated with *CMD1* or *CMD2*-associated resistance in cassava has not been identified. It is suggested that SACMV Rep or CP encoded by the *AC1* or *AV1* gene, respectively, on DNA A could be candidate effectors for NLR-mediated resistance; however, further studies are warranted. Further research on the effect of *MeRPPL1* expression knockdown on the quantification of protein translation also needs to be determined from in planta studies. Furthermore, molecular mechanisms/responses in plants, such as resistance, tolerance and biotic stress, often display similar molecular signatures/features, and in perennial crops, such as cassava, it has been proposed that CMD tolerance may also be associated with biotic stress responses [48].

Nucleotide mutations induced by CRISPR/Cas9 vector and SACMV, singly or together, led to mutations in the MeRPPL1 protein in treated TME3 protoplasts, resulting in about 40–90% deduced amino acid alterations in MeRPPL1 peptides (Figure 4). Such mutations may have affected the detection of SACMV DNA-derived effectors by the MeRPPL1 protein or reduced its functionality, possibly leading to an attenuated or delayed ETI defence response. This would have led to increased SACMV-DNA A replication, which was observed in this study. Similar results in different hosts have been reported, where geminiviruses co-infected with CRISPR vectors induced random mutations in targeted genes/proteins, resulting in a loss of gene function. Some examples include studies on TYLCV in tomato [49,50], SACMV in cassava [30], and cotton leaf curl geminiviruses in cotton [51]. A previous study in TME3 protoplasts also revealed that SACMV DNA accumulation induced multiple mutations in a *MeE3L* homolog [30]. Resistance genes have been shown to be highly susceptible to mutations caused by viral and bacterial pathogens [49,50,51,52]. For example, *Potato virus X* induced random mutations in twenty-five CC-NLR R genes in potato [52]. It was also demonstrated in this study that the disruption of the central nucleotide-binding (NBS) domain’s highly conserved and strictly ordered MHD motif in the *Rx* R gene occurred. Notably, mutations caused by SACMV also resulted in several amino acid alterations in the MHD motif of the MeRPPL1 protein in CRISPR vectors + SACMV and SACMV-only treated protoplasts (Figure 4B). The MHD motif is found in the ARC2 region of the NB-ARC domain. It functions in nucleotide-dependent conformational changes, which lead to the exchange of ADP for ATP and the subsequent activation of the defence mechanisms [53]. Mutations in this motif have been linked to the auto-activation of many NLR proteins in the absence of a viral effector [54,55,56,57]. The histidine (H) residue was noted to be crucial to the functioning of this motif in transducing pathogen perception by LRR [58,59]. In this study, histidine was substituted with tyrosine (Y) in RCS treatments, and cysteine (C) in S-treated TME3 protoplasts (Figure 4B), suggesting that these substitutions could potentially affect the functioning of this MeRPPL1 protein as a receptor, hence disrupting further downstream defence responses. This is not the first report of the disruption of the MHD motif in R proteins in cassava TME3 landrace. It was previously reported that SACMV-induced amino acid substitutions in planta resulted in the disruption or total removal of the MHD motif in some R gene accessions [28]. Further, mutations in R proteins could lead to alterations in the predicted secondary structure, which would affect the conformation of the protein and how the protein interacts with other cellular proteins and molecules. Herein, mutations were shown to affect the secondary structure of the MeRPPL1 predicted proteins in TME3, as illustrated in Figure 5. From these results, it is proposed that in TME3 protoplasts, the mutations induced by CRISPR and SACMV in the MHD motif and other regions of the *MeRPPL1* gene cause secondary structure alterations of MeRPPL1 proteins which would have disrupted binding of SACMV effector proteins or binding and interactions with other cellular proteins involved in the activation of signal pathways for defence. Further studies on the effects of protein alterations would prove informative.

## 5. Conclusions

The CRISPR/Cas9 system successfully induced the gene expression knockdown of *MeRPPL1* in TME3 protoplasts. CRISPR-mediated introduction of random point mutations or deletions resulted in polypeptide amino acid alterations in the MeRPPL1 protein. We demonstrate that these two events contribute to an increase in SACMV DNA replication. Therefore, we conclude that this CC-NLR (CNL) gene, *MeRPPL1*, plays a role in cassava TME3 tolerance to SACMV. Amino acid alterations in the highly conserved MHD motif in MeRPPL1 proteins may have hindered the recognition and binding of the SACMV effector due to alterations in the predicted secondary structure of the MeRPPL1 protein. Thus, the activation of downstream signalling and defence pathways would be disrupted, leading to high SACMV-DNA A replication in TME3 protoplasts. Further identification of the SACMV effector, and in planta studies, would prove informative. Investigation into the intersection between innate immunity (PTI), R protein triggering of ETI, and biotic stress is warranted and would reveal some clues for the future management and engineering of resistance to geminiviruses in crops.

## Data Availability

All relevant data are available in the manuscript.

## Supplementary Materials

The following supporting information can be downloaded at: https://www.mdpi.com/article/10.3390/v16060941/s1, Figure S1: CRISPR-Cas9 gRNA cassette for *MeRPPL1* knockdown; Figure S2: pC1380-*Cas9* vector; Figure S3: Map of *MeRPPL1* (Manes.12G091600) locus (red text) on chromosome 12 of the *Manihot esculenta* v8.1 genome; Figure S4: Predicted per-residue confidence metric (pLDDT) and Predicted Aligned Error of MeRPPL1 protein from references TME3/AM560-2; Figure S5: Predicted per-residue confidence metric (pLDDT) and Predicted Aligned Error of MeRPPL1 protein from TME3 RC sample; Figure S6: Predicted per-residue confidence metric (pLDDT) and Predicted Aligned Error of MeRPPL1 protein from TME3 RCS sample; Figure S7: Predicted per-residue confidence metric (pLDDT) and Predicted Aligned Error of MeRPPL1 protein from TME3 S sample.
