# Supplementary material for: A Coiled-Coil Nucleotide-Binding Domain Leucine-Rich Repeat Receptor Gene MeRPPL1 Plays a Role in the Replication of a Geminivirus in Cassava
Source: Viruses. 2024 Jun 11;16(6):941. doi: 10.3390/v16060941 (PMC11209366; doi:10.3390/v16060941)
Supplement: Supplementary file 1 [file viruses-16-00941-s001.zip › viruses-2857357-supplementary.pdf]

Supplementary Materials

Aag ctt CTT TTT TTC TTC TTC TTC GTT CAT ACA GTT TTT TTT TGT TTA TCA GCT TAC ATT TTC TTG AAC
CGT AGC TTT CGT TTT CTT CTT TTT AAC TTT CCA TTC GGA GTT TTT GTA TCT TGT TTC ATA GTT TGT CCC
AGG ATT AGA ATG ATT AGG CAT CGA ACC TTC AAG AAT TTG ATT GAA TAA AAC ATC TTC ATT CTT AAG ATA
TGA AGA TAA TCT TCA AAA GGC CCC TGG GAA TCT GAA AGA AGA GAA GCA GGC CCA TTT ATA TGG GAA AGA
ACA ATA GTA TTT CTT ATA TAG GCC CAT TTA AGT TGA AAA CAA TCT TCA AAA GTC CCA CAT CGC TTA GAT
AAG AAA ACG AAG CTG AGT TTA TAT ACA GCT AGA GTC GAA GTA GTG ATT G cc atg ggA ACA GAG CAC
CAG TGG TCT AGT GGT AGA ATA GTA CCC TGC CAC GGT ACA GAC CCG GGT TCG ATT CCC GGC TGG
TGC A GG ACT ATG AAT TCC ATA ACT gtt tta gag cta gaa aat agc aag tta aaa taa ggc tag tcc gtt atc aac
ttg aaa aag tgg cac cga gtc ggt gc *g* ggA ACA GAG CAC CAG TGG TCT AGT GGT AGA ATA GTA CCC TGC
CAC GGT ACA GAC CCG GGT TCG ATT CCC GGC TGG TGC A CA AAC TTT ACA ATT ATG TGT gtt tta gag
cta gaa aat agc aag tta aaa taa ggc tag tcc gtt atc aac ttg aaa aag tgg cac cga gtc ggt gc***t ttt tt*** a gat ct

KEY:
Arabidopsis U6-26 Promoter
Pre-tRNA
gRNA
SpCas9 tracrRNA (scaffold)
terminator

Figure S1. CRISPR-Cas9 gRNA cassette for *MeRPPL1* knockdown. Two gRNAs (in back text), identified from CRISPOR version 4.7 were used to design a duplex single gRNA (sgRNA) targeting *MeRPPL1.* The sgRNAs were synthesised in a pBluescript II SK (+) plasmid by Inqaba Biotechnology Industries (Pretoria, SA) and digested out using restriction enzymes HindIII (aagctt) and BglIII (agatct).

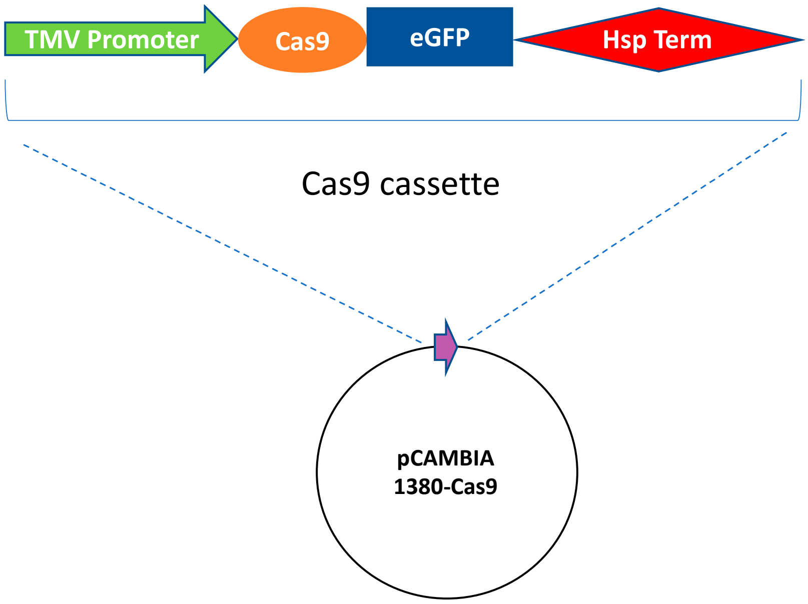

Figure S2 pC1380-*Cas9* silencing vector: the vector comprises a promoter from tobacco mosaic virus (TMV), Cas9 protein (obtained from the bacteria *Streptococcus pyogenes*) functions together with a “guide” RNA that targets a complementary 20-nucleotide stretch of DNA. Once the RNA identifies a sequence matching these nucleotides, Cas9 cuts the double-

stranded DNA helix. eGFP= enhanced green fluorescent protein. HSP = heat shock protein 18.2 terminator that increases mRNA levels of both transiently and stably expressed transgenes approximately 2-fold more than the NOS (nopaline synthase) terminator.

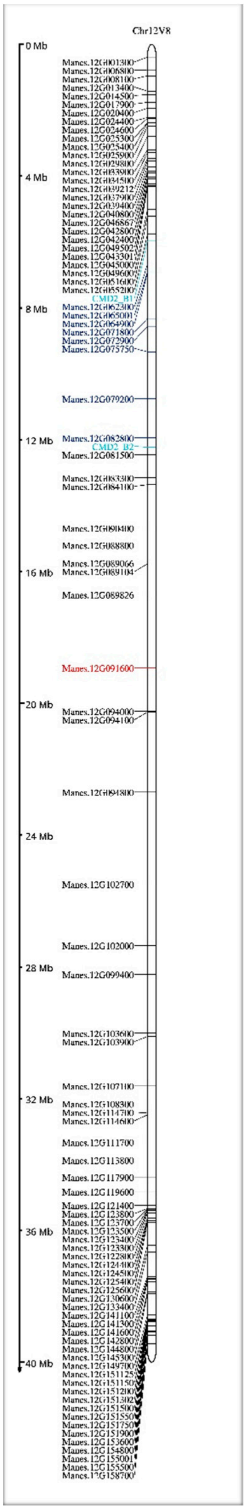

Figure S3 Map of *MeRPPL1* (Manes.12G091600) locus (red text) on chromosome 12 of the *Manihot esculenta* v8.1 genome.

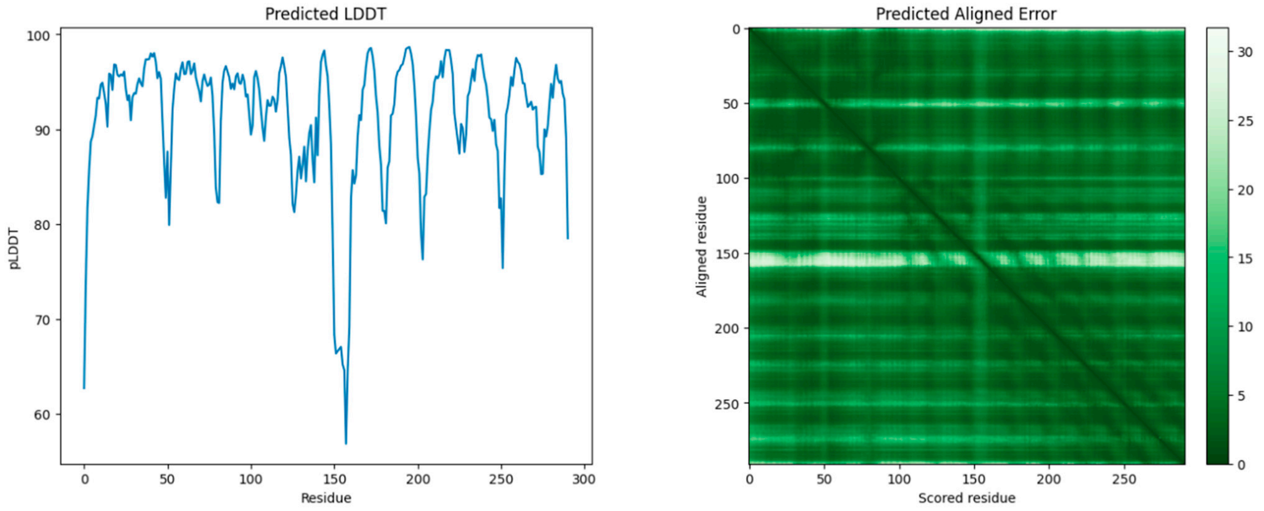

Figure S4. Predicted per-residue confidence metric (pLDDT) and Predicted Aligned Error of MeRPPL1 protein from references TME3/AM560-2. pLDDT corresponds to the model's predicted score and is used to colour-code the residues of the predicted model in the 3D structure viewer [1]. pLDDT > 90 are expected to be modelled to high accuracy; between 70 and 90 are expected to be modelled well; between 50 and 70 are low confidence; less than 50 is a reasonably strong predictor of disorder. Predicted Aligned Error (PAE) assesses confidence in the domain packing and large-scale topology of the protein. The colour at (x, y) indicates AlphaFold's expected position error at residue x if the predicted and true structures were aligned on residue y. If the PAE is low it indicates that AlphaFold predicts well-defined relative positions and orientations for them; if the PAE is high the relative positions and/or orientations of these domains in the 3D structure are uncertain.

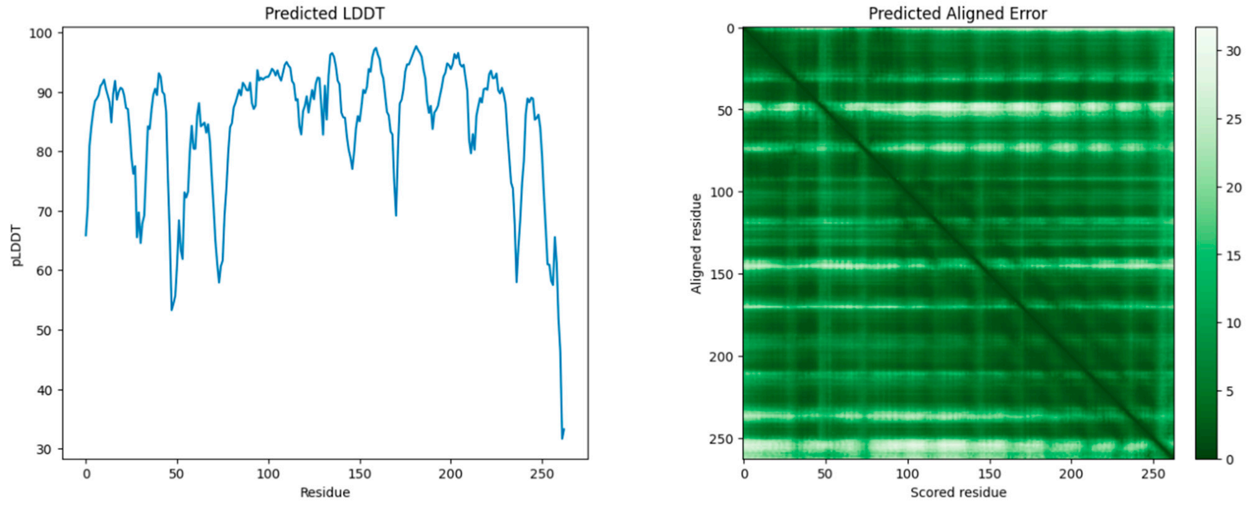

Figure S5. Predicted per-residue confidence metric (pLDDT) and Predicted Aligned Error of MeRPPL1 protein from TME3 RC sample. pLDDT corresponds to the model's predicted score and is used to colour-code the residues of the predicted model in the 3D structure viewer [1]. pLDDT > 90 are expected to be modelled to high accuracy; between 70 and 90 are expected to be modelled well; between 50 and 70 are low confidence; less than 50 is a reasonably strong predictor of disorder. Predicted Aligned Error (PAE) assesses confidence in the domain packing and large-scale topology of the protein. The colour at (x, y) indicates AlphaFold's expected position error at residue x if the predicted and true structures were aligned on residue y. If the PAE is low it indicates that AlphaFold predicts well-defined relative positions and orientations for them; if the PAE is high the relative positions and/or orientations of these domains in the 3D structure are uncertain.

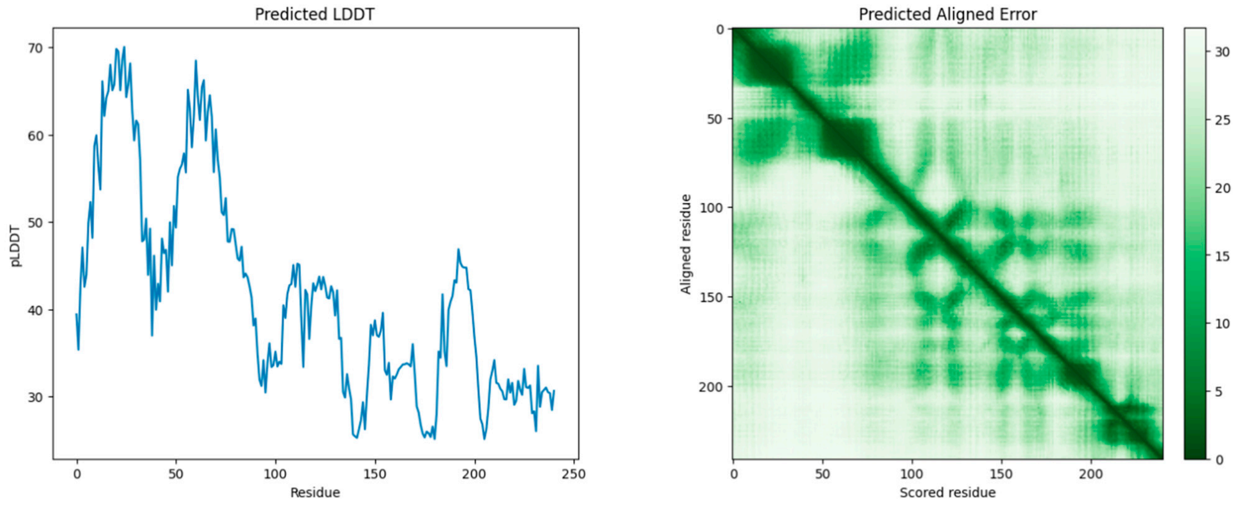

Figure S6. Predicted per-residue confidence metric (pLDDT) and Predicted Aligned Error of MeRPPL1 protein from TME3 RCS sample. pLDDT corresponds to the model's predicted score and is used to colour-code the residues of the predicted model in the 3D structure viewer [1]. pLDDT > 90 are expected to be modelled to high accuracy; between 70 and 90 are expected to be modelled well; between 50 and 70 are low confidence; less than 50 is a reasonably strong predictor of disorder. Predicted Aligned Error (PAE) assesses confidence in the domain packing and large-scale topology of the protein. The colour at (x, y) indicates AlphaFold's expected position error at residue x if the predicted and true structures were aligned on residue y. If the PAE is low it indicates that AlphaFold predicts well-defined relative positions and orientations for them; if the PAE is high the relative positions and/or orientations of these domains in the 3D structure are uncertain.

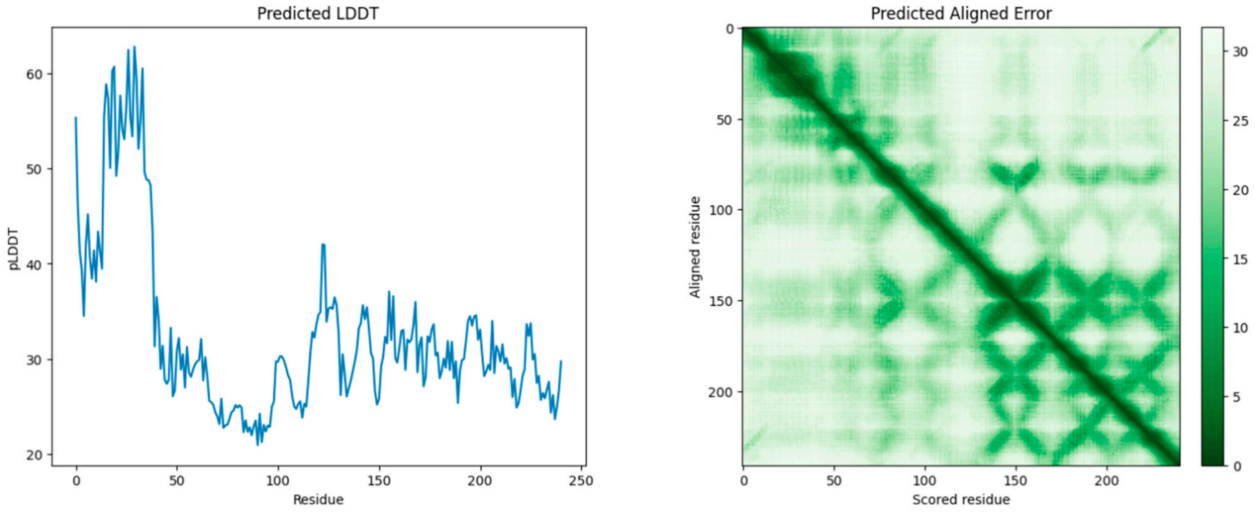

Figure S7. Predicted per-residue confidence metric (pLDDT) and Predicted Aligned Error of MeRPPL1 protein from TME3 S sample. pLDDT corresponds to the model's predicted score and is used to colour-code the residues of the predicted model in the 3D structure viewer [1]. pLDDT > 90 are expected to be modelled to high accuracy; between 70 and 90 are expected to be modelled well; between 50 and 70 are low confidence; less than 50 is a reasonably strong predictor of disorder. Predicted Aligned Error (PAE) assesses confidence in the domain packing and large-scale topology of the protein. The colour at (x, y) indicates AlphaFold's expected position error at residue x if the predicted and true structures were aligned on residue y. If the PAE is low it indicates that AlphaFold predicts well-defined relative positions and

orientations for them; if the PAE is high the relative positions and/or orientations of these domains in the 3D structure are uncertain

References

1. Mariani, V.; Biasini, M.; Barbato, A.; Schwede, T. LDDT: A Local Superposition-Free Score for Comparing Protein Structures and Models Using Distance Difference Tests. *Bioinformatics* **2013**, *29*, 2722, doi:10.1093/BIOINFORMATICS/BTT473.
